# A Multilocus Genetic Study in a Cohort of Italian SLE Patients Confirms the Association with STAT4 Gene and Describes a New Association with HCP5 Gene

**DOI:** 10.1371/journal.pone.0111991

**Published:** 2014-11-04

**Authors:** Cinzia Ciccacci, Carlo Perricone, Fulvia Ceccarelli, Sara Rufini, Davide Di Fusco, Cristiano Alessandri, Francesca Romana Spinelli, Enrica Cipriano, Giuseppe Novelli, Guido Valesini, Paola Borgiani, Fabrizio Conti

**Affiliations:** 1 Department of Biomedicine and Prevention, Section of Genetics, School of Medicine, University of Rome “Tor Vergata”, Rome, Italy; 2 Lupus Clinic, Reumatologia, Dipartimento di Medicina Interna e Specialità Mediche, Sapienza Università di Roma, Rome, Italy; Beth Israel Deaconess Medical Center, United States of America

## Abstract

**Background:**

Systemic lupus erythematosus (SLE) is an autoimmune disease with complex pathogenesis in which genes and environmental factors are involved. We aimed at analyzing previously identified loci associated with SLE or with other autoimmune and/or inflammatory disorders (STAT4, IL10, IL23R, IRAK1, PSORS1C1, HCP5, MIR146a, PTPN2, ERAP1, ATG16L1, IRGM) in a sample of Italian SLE patients in order to verify or confirm their possible involvement and relative contribution in the disease.

**Materials and methods:**

Two hundred thirty-nine consecutive SLE patients and 278 matched healthy controls were enrolled. Study protocol included complete physical examination, and clinical and laboratory data collection. Nineteen polymorphisms were genotyped by allelic discrimination assays. A case-control association study and a genotype-phenotype correlation were performed.

**Results:**

STAT4 was the most associated gene [P = 3×10^−7^, OR = 2.13 (95% CI: 1.59–2.85)]. IL10 confirmed its association with SLE [rs3024505: P = 0.02, OR = 1.52 (95% CI: 1.07–2.16)]. We describe a novel significant association between HCP5 locus and SLE susceptibility [rs3099844: P = 0.01, OR = 2.06 (95% CI: 1.18–3.6)]. The genotype/phenotype correlation analysis showed several associations including a higher risk to develop pericarditis with STAT4, and an association between HCP5 rs3099844 and anti-Ro/SSA antibodies.

**Conclusions:**

STAT4 and IL10 confirm their association with SLE. We found that some SNPs in PSORS1C1, ATG16L1, IL23R, PTPN2 and MIR146a genes can determine particular disease phenotypes. HCP5 rs3099844 is associated with SLE and with anti-Ro/SSA. This polymorphism has been previously found associated with cardiac manifestations of SLE, a condition related with anti-Ro/SSA antibodies. Thus, our results may provide new insights into SLE pathogenesis.

## Introduction

Systemic lupus erythematosus (SLE) is a chronic autoimmune disease characterized by the production of autoantibodies against intracellular, nucleic acid and cell surface antigens. The disease is relapsing and affects multiple organ systems including the skin, kidneys, lungs, heart and the central nervous system. The pathogenesis of SLE is complex, with several susceptibility genes and environmental factors involved in its development and clinical manifestations. Familial aggregation and a higher rate of concordance for SLE in monozygotic than in dizygotic twins [1], [2] provide support for a strong role of genetic factors in the pathogenesis of this disorder.

During last decade, many genes were described for their potential role in predisposing to SLE [3]. The list of involved genes has increased with the advent of Genome Wide Association (GWA) studies. These studies confirmed genetic associations of over 40 genes [3]. The number of genes emphasizes the complexity of the inheritability of SLE. Most of the identified genes encode proteins that participate in key pathogenic pathways, including Toll-like receptor and type I interferon signaling pathways, immune regulation pathways and those that control the clearance of immune complexes [4].

Interestingly, SLE and other autoimmune diseases share many of the risk loci identified, suggesting the involvement of common pathways in autoimmune diseases [5]. Moreover, the existence of common genetic susceptibility was already suggested by the clustering of multiple autoimmune diseases within families, including families with SLE [2], [6]. This genetic overlap is exemplified by the well-known associations of certain HLA loci with multiple autoimmune disease as well as non-HLA risk loci in different pathways such as IL2RA, STAT4, PTPN22 and IFIH1 [7], [8]. In this context, in a previous paper, we have described for the first time, an association between SLE and the TRAF3IP2 gene [9], a gene involved in psoriasis and psoriatic arthritis [10], but also described in cutaneous complications in inflammatory bowel diseases [11].

Here, our aim was to analyze a number of previously identified loci associated with SLE or with other autoimmune and/or inflammatory diseases (STAT4, IL10, IL23R, IRAK1, PSORS1C1, HCP5, MIR146a, PTPN2, ERAP1, ATG16L1, IRGM) in a sample of Italian SLE patients in order to confirm or verify their possible involvement and their relative contribution in SLE susceptibility. The genes were chosen due their role in different pathways involved in SLE pathogenesis. We therefore performed a case/control study and a genotype-phenotype correlation analysis.

## Materials and Methods

### Sample collection

Two hundred and thirty-nine SLE consecutive Caucasian patients ≥18 years of age affected with SLE were retrospectively enrolled at the Lupus Clinic of the Rheumatology Unit, Sapienza University of Rome (Sapienza Lupus Cohort). SLE diagnosis was performed according to the revised 1997 American College of Rheumatology (ACR) criteria. This cohort was already described in Perricone et al. 2013 [9]. Briefly, 21 (8.8%) were males and 218 (91.2%) females, mean age ± SD was 42.7±11.4 years, mean age at diagnosis ±SD was 30.9±11.6 years. Musculo-skeletal was the most frequent involvement (71.0% of patients), 96.6% had positive ANA, and 73.3% had positive anti-dsDNA. Study protocol included complete physical examination and blood drawing. The clinical and laboratory data were collected in a standardized, computerized, electronically-filled form including demographics, past medical history with date of diagnosis, co-morbidities, and previous and concomitant treatments.

The evaluation of clinical and laboratory parameters was performed at the Lupus Clinic of Sapienza University of Rome. Clinical and laboratory features were assessed with a dichotomous score (present = 1; absent = 0).

Written informed consent was obtained from each patient and the ethic committee of Sapienza University of Rome approved the study design.

Two hundred seventy-eight age- and ethnicity-matched healthy subjects, enrolled at University of Rome Tor Vergata, served as controls.

### Clinical laboratory analysis

Among the laboratory tests, the following parameters were evaluated: complete blood count [CBC], including erythrocytes, leucocytes and platelets count, erythrocyte sedimentation rate [ESR], C-reactive protein [CRP] levels, serum levels of creatinine and complement C3 and C4, anti-nuclear (ANA), anti-double-stranded DNA (anti-dsDNA), ENA, anti-Sm, anti-RNP, anti-Ro/SSA, anti-La/SSB, anti-CL IgG and IgM, anti-β_2_GPI IgG and IgM, and LA. To be positive, each of these tests had to be positive in at least 2 occasions 12 weeks apart all along patient clinical history.

ANA were determined by means of indirect immunofluorescence (IIF) on Hep-2 (titer ≥1:160 or ++ on a scale from + to ++++); anti-dsDNA with ELISA assays (considering levels higher than the cut-off of the reference laboratory) or IIF on Crithidia Luciliae, ENA (including anti-Ro/SSA, anti-La/SSB, anti-Sm, anti-RNP) by ELISA assay considering titers above the cut-off of the reference laboratory, anti-CL of IgG or IgM isotype, by ELISA, in serum or plasma at medium or high titers [e.g.,>40 standardized international units (GPL/MPL units) or above the 99^th^ percentile], anti-β_2_GPI of IgG or IgM isotype, by ELISA, in serum or plasma (above the 99^th^ percentile), and finally LAC was assessed according to the guidelines of the International Society on Thrombosis and Hemostasis (Scientific Subcommittee on lupus anticoagulant/phospholipid-dependent antibodies) [12].

All subjects underwent blood drawing (5 ml supplemented with 0.5% EDTA) in order to perform genetic analysis.

### DNA extraction and genotyping

Genomic DNA was isolated from peripheral blood mononuclear cells using a Qiagen blood DNA mini kit. A panel of SNPs was selected on the basis of literature data in 11 genes involved in immune response, autophagy and inflammation, already described as associated with any kind of autoimmune disease. The selected SNPs were the following: rs7574865 (STAT4), rs1800872 and rs3024505 (IL10), rs9469003 and rs3099844 (HCP5), rs11209026, rs11803505 and rs7517847 (IL23R), rs2233945 (PSORS1C1), rs13361189 and rs4958847 (IRGM), rs2241880 (ATG16L1), rs2542151 and rs7234029 (PTPN2), rs30187 and rs27524 (ERAP1), rs2910164 and rs2431697 (MIR146A) and rs3027898 (IRAK1). Genotyping was performed by allelic discrimination assay by TaqMan assays (Applied Biosystems, Foster City, CA, USA) and ABI PRISM 7000. Each assay was run with positive (samples previously confirmed by direct sequencing as heterozygous and/or variant homozygous) and negative controls.

### Statistical analysis

The Hardy–Weinberg equilibrium was verified for all SNPs by the Pearson χ^2^ test.

Differences in alleles/genotypes/haplotypes frequencies between cases and control were evaluated by Pearson χ^2^ test. Haplotypes were inferred using Arlequin, version 3.11 [13]. Odds ratios (ORs) with 95% CI were calculated. A genotype-phenotype correlation analysis has been performed considering the heterozygotes and variant homozygotes together (1degree of freedom [df]). A binary logistic regression analysis (stepwise) was carried out considering the presence/absence of SLE as dependent variable and all genetics associated factors as independent variables. All statistical analyses were performed by the SPSS 13.0 program (SSPS Inc., IL, USA).

## Results

### Case/control association analysis

We studied 19 polymorphisms in 11 genes. Deviations from Hardy–Weinberg equilibrium were not observed. In Table 1, we reported the comparisons of the allele frequencies between cases and controls for all studied polymorphisms. Among the studied genes, STAT4 resulted, as expected, the most associated with a P = 3×10^−7^ and OR = 2.13 (95% CI 1.59–2.85). Moreover, the Mantel–Haenszel test for linear association highlights that there was an additive effect of STAT4 risk allele (P = 5.3×10^−7^): subjects with one risk allele presented an OR = 1.57, while subjects with two risk alleles reached an OR = 8.4 (95% CI 3.42–20.72, Table 2). Furthermore, IL10 confirmed the association with SLE in our cohort with the rs3024505 SNP (P = 0.02 and OR = 1.52 [95% CI 1.07–2.16]).

**Table 1:** Results of allelic association analysis.

Gene	SNP	SLE MAF*%	Controls MAF%	P	OR
**STAT4**	rs7574865	0.35	0.2	3×10^−7^	2.13 (1.59–2.85)
**IL10**	rs1800872	0.26	0.31	0.09	0.79 (0.6–1.04)
	rs3024505	0.17	0.12	0.02	1.52 (1.07–2.16)
**HCP5**	rs9469003	0.18	0.19	0.67	0.93 (0.65–1.32)
	rs3099844	0.1	0.05	0.01	2.06 (1.18–3.6)
**IL23R**	rs11209026	0.053	0.078	0.1	0.66 (0.4–1.09)
	rs11803505	0.34	0.34	1	1 (0.77–1.31)
	rs7517847	0.4	0.39	0.65	1.06 (0.82–1.87)
**PSORS1C1**	rs2233945	0.2	0.17	0.26	1.19 (0.87–1.63)
**IRGM**	rs13361189	0.09	0.11	0.38	0.79 (0.46–1.34)
	rs4958847	0.13	0.16	0.43	0.84 (0.54–1.3)
**ATG16L1**	rs2241880	0.46§	0.43§	0.39	0.87 (0.64–1.19)
**PTPN2**	rs2542151	0.14	0.11	0.38	1.29 (0.73–2.3)
	rs7234029	0.13	0.15	0.48	0.81 (0.45–1.46)
**ERAP1**	rs30187	0.36	0.38	0.65	0.93 (0.67–1.28)
	rs27524	0.38	0.38	0.89	1.02 (0.79–1.32)
**MIR146A**	rs2910164	0.3	0.27	0.49	1.1 (0.84–1.45)
	rs2431697	0.41	0.42	0.63	0.93 (0.68–1.26)
**IRAK1**	rs3027898	0.29	0.26	0.3	1.17 (0.87–1.6)

* MAF: Minor allele frequency,

§ Allele A.

**Table 2:** Genotypes distribution for rs7574865 STAT4 SNP revealed an allele dose additive effect.

	SLE Patients	Controls			*Mantel–Haenszel test of linearity*
	n = 234	n = 243	P	OR (95% CI)	
**GG**	104	150			5.3×10^−7^
**GT**	95	87	0.02	1.57 (1.07–2.31)	
**TT**	35	6	2.4×10^−7^	8.4 (3.42–20.72)	

GT+TT *vs* GG: P = 0.00016, OR = 2 (1.4–2.9).

Moreover, for the first time, we described an association between HCP5 locus and SLE susceptibility: the variant allele of rs3099844 resulted a risk allele with P = 0.01 and OR = 2.06 (95% CI 1.18–3.6). No significant associations were observed for all the other studied SNPs.

A binary logistic regression analysis was carried out considering the three associated SNPs as independent variables and the presence/absence of SLE as dependent variable (Table 3). This analysis confirmed the involvement of STAT4 and HCP5. This model explains the 5.9% of genetic susceptibility (Cox and Snell R^2^).

**Table 3:** Final model of binary logistic regression analysis (stepwise method) considering only associated genetic factors.

	B	P	OR (95%CI)	R^2^ (Cox & Snell)
**STAT4_rs7574865**	0.931	<0.0001	2.54 (1.58–4.06)	0.059
**HCP5_rs3099844**	0.835	0.022	2.31 (1.13–4.71)	

### Haplotypes analysis

We inferred the haplotypes distribution for IL10, IL23R, HCP5, IRGM, PTPN and ERAP1 genes. However, we did not find any significant association comparing the distribution in cases and controls (data not shown). Only for IL10 we observed a significant difference comparing the TC (composed by the variant allele of rs3024505 and wildtype allele of rs1800872) *vs* CA (composed by the wild-type allele of rs3024505 and the variant allele of rs1800872) haplotype (P = 0.012 and OR = 1.7 [95% CI 1.12–2.56]), but this evidence does not improve consistently the statistical significance obtained considering only the rs3024505 SNP.

### Number of risk alleles

**Figure 1:**
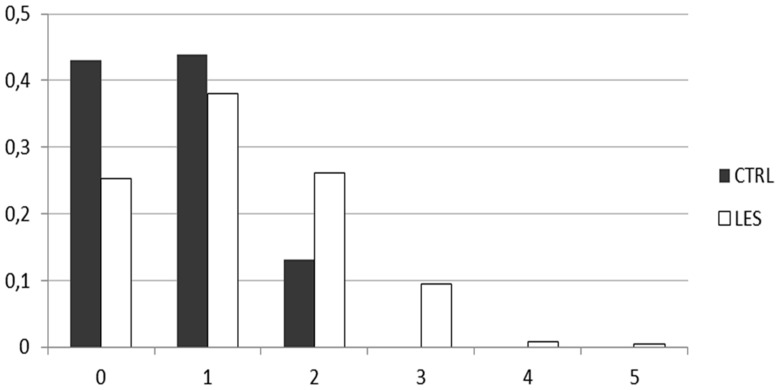
Counting of risk alleles in cases and controls. The risk alleles considered are the variant alleles of rs7574865, rs3099844 and rs3024505 SNPs.

We counted the number of risk alleles, considering as risk alleles the variant alleles of rs7574865 (STAT4), rs3024505 (IL10), and rs3099844 (HCP5) SNPs, and we evaluated the different risk allele number distribution between cases and controls (Figure 1, Table 4). As expected the class with no risk allele was significantly more present in controls than in patients (P = 0.0008, OR = 0.45). On the contrary, the class with two risk alleles was significantly more present in cases than in controls (P = 0.0003, OR = 3.78). Finally, genetic profiles with three or more risk alleles were present only in SLE patients (P<0.0001).

**Table 4:** Counting of risk alleles in cases and controls.

Number of risk alleles	Controls	SLE patients	Comparisons	P	OR
0	49	59	0 *vs* others	0.0008	0.45 (0.28–0.72)
1	50	89	1 *vs* 0	0.13	1.47 (0.88–2.47)
2	15	61	2 vs 0	0.0003	3.78 (1.71–6.67)
3		22	(3+4+5) *vs* 0	<0.0001	ND
4		2			
5		1			
Total	114	234			

P_tot_ = 8*10^−6^.

(3df).

The risk alleles considered are the variant alleles of rs7574865, rs3099844 and rs3024505 SNPs.

### Genotype-phenotype correlation analysis

A genotype/phenotype correlation analysis between the genotypes and clinical characteristics of SLE patients was performed. Results of this analysis were reported in Table 5. STAT4, that showed the strongest association with the disease, was also associated with a higher risk to develop pericarditis, while HCP5 SNPs resulted associated with anti-Ro/SSA, anti-dsDNA and aCL.

**Table 5:** Association between disease phenotypes and studied polymorphisms.

Phenotype	Gene	Locus	P	OR (95% CI)
**Pericarditis**	ATG16L1	rs2241880	0.024	0.43 (0.2–0.9)
	STAT4	rs7574865	0.015	2.32 (1.17–4.6)
	PTPN2	rs2542151	0.032	2.35 (1.07–5.18)
**Serositis**	PTPN2	rs2542151	0.022	2.49 (1.13–5.5)
**Aphthous/ulcers**	IL23R	rs7517847	0.046	2.13 (1.0–4.52)
	IL23R	rs11209026	0.045	2.6 (0.99–6.86)
**Arthritis**	MIR146A	rs2910164	0.024	1.93 (1.09–3.4)
**Malar Rash**	IL23R	rs11209026	0.048	0.44 (0.19–1.01)
**aCL**	IL10	rs3024505	0.027	0.49 (0.26–0.93)
	HCP5	rs3099844	0.005	0.34 (0.15–0.74)
	Mir146a	rs2431697	0.035	2.03 (1.05–3.95)
**ENA**	STAT4	rs7574865	0.018	1.93 (1.12–3.36)
**C3 (below normal values)**	MIR146A	rs2910164	0.029	1.91 (1.06–3.4)
**Anti-Ro/SSA**	PSORS1C1	rs2233945	0.024	0.48 (0.25–0.91)
	HCP5	rs9469003	0.007	0.37 (0.18–0.78)
	HCP5	rs3099844	0.016	2.28 (1.15–4.52)
**LAC**	PSORS1C1	rs2233945	0.008	2.44 (1.25–4.76)
**C4 (below normal values)**	PSORS1C1	rs2233945	0.009	2.24 (1.22–4.12)
**Anti-dsDNA**	HCP5	rs3099844	0.047	0.5 (0.25–1)

Interestingly, some SNPs that did not associate with the disease, showed association with particular disease phenotypes. For example, PTPN2 SNP resulted associated with a higher risk to develop pericarditis and serositis, while ATG16L1 SNP resulted protective towards pericarditis. IL23R SNPs resulted associated with a higher risk to develop aphthous/ulcer and with a minor risk to develop malar rash. MIR146A SNP resulted associated with a higher risk to develop arthritis and with C3 consumption. PSORS1C1 resulted associated with the presence of LAC and decreased C4.

Moreover, PSORS1C1 resulted also associated with age at diagnosis (table 6): indeed the average age at onset is significantly lower in patients with rs2233945 SNP variant (28.6 vs 32.2 years, P = 0.042).

**Table 6:** PSORS1C1 SNP correlates with age at onset.

	Mean Age at diagnosis	N	Standard Deviation	P
CC	32.19	124	11.46	0.042
CA+AA	28.65	69	11.57	

## Discussion

In the last years, the advent of genome wide association studies and the technological possibility to detect polymorphisms in large samples have revolutionized the field of human genetics by identification and confirmation of common gene variants that confer susceptibility to a plethora of diseases. A number of studies support the concept that common risk genes underlie multiple autoimmune disorders and suggest the involvement of common pathways of pathogenesis among different diseases [14]. In this study, we have examined specific genes, involved in autoimmunity, in inflammation and in autophagy to evaluate their involvement in SLE susceptibility. We aimed at confirming the association between STAT4, IL10 and IRAK1 [15]–[19] in a monocentric Italian cohort with SLE. Other genes, such as IL23R, PSORS1C1, HCP5, were found to be associated with psoriasis and rheumatoid arthritis [20]–[23]. Regarding genes involved in autophagy, we have chosen IRGM and ATG16L1 as candidate genes, since they were associated with Crohn's disease [24]. PTPN2 was associated with Crohn's disease [25] and type I diabetes [26]; ERAP1 was associated with psoriasis [27] and multiple sclerosis [28].

In our study, only three genes resulted associated with the disease development: STAT4, IL10 and HCP5. Indeed, we confirmed the STAT4 rs7574865 variant allele as a risk factor for SLE susceptibility, with an OR even higher than previously described (OR = 2.13 [95% CI 1.59–2.85]). Moreover, we observed an allele-dose risk effect, whereby individuals with two STAT4 alleles have 8.4-fold risk to develop the disease compared with wild-type subjects, while heterozygous subjects show 1.57-fold risk. Considering the fact that, usually, each risk factor gives a moderate contribution to SLE susceptibility, in our cohort, this SNP has a considerable weight. STAT4 is a member of the STAT family of transcription factors, and is involved in mediating responses to IL12 in lymphocytes, and regulating the differentiation of T helper cells. The STAT4 rs7574865 polymorphism, located within the third intron of the gene, shows the strongest association with several autoimmune conditions [29], [30], such as SLE, RA, type 1 and 2 diabetes, systemic sclerosis, inflammatory bowel diseases, primary Sjogren's syndrome (SS), juvenile idiopathic arthritis, primary antiphospholipid syndrome, autoimmune thyroid diseases, multiple sclerosis, psoriasis, granulomatosis with polyangiitis, giant cell arteritis [31].

The STAT4 gene can be activated and phosphorylated upon ligation of the type I IFN receptor by IFN-α, and subsequently induce downstream transcription of IFN-α-induced genes [32]. It has been recently showed that a great number of genes target for STAT4 are enriched to functional pathways in the type I interferon system, and have key roles in the inflammatory response. Thus, by participating in transcription complex with other co-factors, STAT4 harbors the potential of regulating a large number of target genes, which may contribute to their strong association with SLE [33].

The other interesting result is the association between a gene variant in HCP5 gene and SLE susceptibility that was never described before. Indeed, this is the first study, by our knowledge, that evaluated the involvement of this gene in SLE susceptibility. Moreover, HCP5 SNPs resulted associated with anti-Ro/SSA and anti-dsDNA antibodies. The rs3099844 polymorphism has been previously described as involved in Steven Johnson Syndrome and Toxic Epidermal Necrolysis susceptibility [34]–[36], but also in another autoimmune condition, primary sclerosing cholangitis [37]. In this condition, anti-ENA including anti-SSA can be found in up to 11.5% of patients and anti-dsDNA at a lesser extent (1–3% of patients). However, the most interesting study is a genome-wide association study showing an association between the rs3099844 and the cardiac manifestations of systemic lupus erythematosus [38]. As it is well known, this clinical condition is associated with the presence of anti-Ro/SSA antibodies [39]–[43]. Altogether, these data suggest a strong evidence of an association of such polymorphism with the anti-Ro/SSA antibodies, and further studies are awaited to clarify the specific underlying mechanisms. Another SNP in the HCP5 gene (rs2395029) was previously associated with psoriasis and psoriatic arthritis [23]. HCP5 gene (major histocompatibility complex P5), located in HLA region, is expressed primarily in cells of the immune system such as spleen, blood and thymus consistent with a potential role in autoimmunity [23]. This allele was associated with lower viral load set point following HIV-1 infection. This is of interest, since anti-Ro/SSA antibodies have been described in HIV infected subjects [44], [45]. Hence, it is possible that HCP5 carriers mount a strong immune reaction to viral infection that, in genetically susceptible individuals, could lead to excessive inflammation. On the other hand, sera from SLE and SS patients were also found to present reactivity with HIV-1 p24 antigen [46], suggesting a specific pattern of nonrandom cross-reactivity between virus p24 and autoimmune sera. Moreover, both a direct interaction between the SSB and HIV-1 trans-activation response element as well as the involvement of the SSB autoantigen in HIV-1 gene expression have been reported previously [47], [48]. Thus, there exist a relationship between the anti-Ro/SSA, anti-La/SSB antibodies and HIV [49] of which HCP5 may represent the bridge.

Finally, besides the confirmation of STAT4 and the newly described association with HCP5, we confirmed the risk effect of IL10 rs3024505 variant allele on SLE development. Polymorphisms in this gene were described as associated with several autoimmune disease, including SLE, rheumatoid arthritis, psoriasis and Crohn's disease, highlighting that IL10 is one of the common risk genes. IL10, primarily produced by monocytes and lymphocytes, is a multifunctional cytokine in immune-regulation and inflammation. A number of studies have been conducted to investigate the association of the IL10 gene polymorphisms with SLE susceptibility, although with conflicting results [18]. Considering the three associated SNPs, we also observed a cumulative genetic effect that distinguish cases from controls (Figure 1). A similar analysis in a previous study, described a different genetic risk scores in males and females [50].

Regarding the other genes, although we did not find any association with the development of the disease, some of the examined polymorphisms resulted associated with particular phenotypes, suggesting that these genes probably do not affect the disease susceptibility but modulate the phenotype. Of course, this lack of association does not mean that these genes are not involved with the disease; indeed, we cannot exclude that other SNPs in these same genes, not investigated in our study, could be associated. Interestingly, PSORS1C1 gene, which maps in the HLA region, resulted associated with LAC, decreased C4, and with an early-onset of the disease. PSORS1C1 gene is one of psoriasis susceptibility loci [51], but it is also associated with systemic sclerosis [52] and has been suggested to play an important role in the development of RA [53]. We also described an association between MiR-146a and the development of arthritis complications. It is known that MiR-146a is a negative regulator of the IFN pathway, and under-expression of MiR-146a conduces to alterations in the type I IFN pathway in lupus patients by targeting the key signaling proteins [54]. Autophagy has been described as a critical homeostatic mechanism in T lymphocytes, influencing proliferation and differentiation [55], and there are studies in the literature which highlighted the association between SLE and autophagy genes [56], [57]. However, in our study, we did not find any association of the disease with ATG16L1 and IRGM genetic variants. Of course, this lack of association could be due to the SNPs selection and other SNPs in the same gene could associate with the disease. Nonetheless, it is interesting to note that rs2241880 in ATG16L1 resulted associated with a minor risk to develop pericarditis as a complication. Admittedly, a limitation of our study is the relatively small number of samples; however, it should be underlined that this a monocentric cohort of patients of the same ethnicity, strictly followed and clinically well characterized.

In conclusion, our study confirms the role of STAT4 and IL10 in SLE susceptibility and describes for the first time the association between a HCP5 genetic variant and SLE susceptibility. These results confirm the importance of the adaptive and innate arms of the immune system in establishing SLE risk. However, each of the identified alleles accounts for only a fraction of the overall genetic risk, and functional studies are necessary to clearly explain their involvement with SLE pathogenesis.
